# A rare case of cervical hypoglossal nerve neurofibromas in a patient with type 1 neurofibromatosis

**DOI:** 10.1016/j.bjorl.2021.01.006

**Published:** 2021-02-25

**Authors:** Sai-Guan Lum, Marina Mat Baki, Mohd Razif Mohamad Yunus

**Affiliations:** a Hospital Canselor Tuanku Muhriz, Universiti Kebangsaan Malaysia Medical Centre, Department of Otorhinolaryngology – Head & Neck Surgery, Kuala Lumpur, Malaysia; b National University of Malaysia, Faculty of Medicine, Kuala Lumpur, Malaysia

## Introduction

Neurofibromas are benign, slow growing tumors that arise from the peripheral nerve sheath. Such tumors are comprised of a mixture of Schwann cells, perineurial cells and fibroblast-like cells.^1^ Approximately 25%–45% of benign nerve sheath tumors occur in the head and neck region, and the majority of these are schwannomas.^2^ Neurofibromas in the cervical region are rare. Most of these neurofibromas (60%–90%) occur in a sporadic fashion, with only 10% of cases associated with Neurofibromatosis type 1 (NF-1), formerly known as von Recklinghausen disease.^2^

Neurofibromatosis type 1 (NF-1) is an autosomal dominant genetic disorder with variable penetrance and expressivity, with an estimated incidence of 1 in 2500 births.^3^ It is associated with a somatic mutation in the NF-1 gene, a tumor suppressor gene located on the long arm of chromosome 17 (17q). Loss of NF-1 gene expression leads to increased levels of activated Rat Sarcoma gene (RAS), which is an oncogene promoting unregulated cell growth and proliferation.^1^ Upregulation of this oncogene results in various pathognomonic features, such as Lisch nodules in the iris, café-au-lait spots, multiple neurofibromas and optic gliomas.

Most previous neurofibromas in the cervical region associated with NF-1 were reported to arise from the vagus nerve.^2^ We describe a rare case of a parapharyngeal neurofibroma originating from the hypoglossal nerve in a patient with NF-1. The tumor was initially thought to arise from the vagus nerve. Radiological imaging features and unexpected intraoperative findings are presented and discussed.

## Case report

A 32-year-old male with underlying NF-1 presented with a bilateral neck mass. He had first noticed the right neck mass 5 years earlier. The mass was painless but progressively enlarging. Two years earlier, he had noticed another swelling on the left side of his neck, similar in characteristics to that of the right neck mass. He also reported recent pressure or discomfort on the right side of his neck and a sensation of a lump on swallowing. He denied any voice change, hearing loss or obstructive symptoms, such as dysphagia or dyspnea. There were no constitutional symptoms such as unexplained weight loss or a recurrent fever. He had a history of excision of a cutaneous lesion on this right forearm and right leg 7 years earlier, with both histopathologically reported as neurofibromas. There was no history of neurofibromatosis in the family.

**Figure 1:**
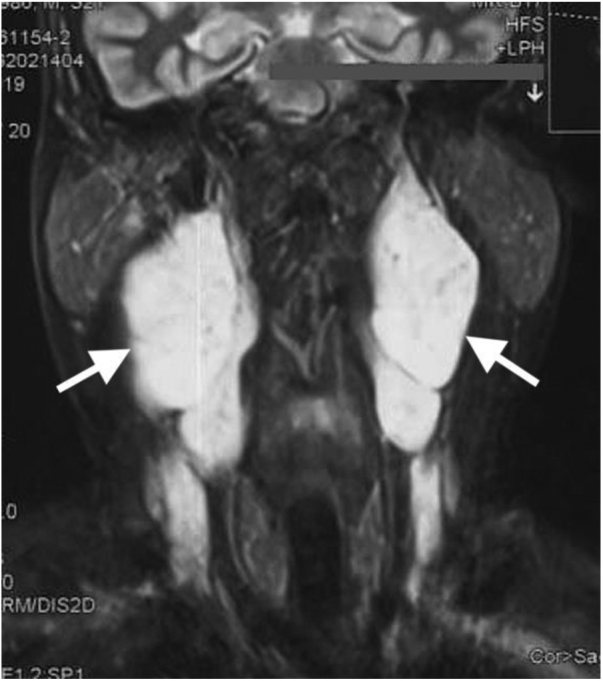
Coronal T2-weighted MRI image shows bilateral hyperintense masses (white arrows) at parapharyngeal space with heterogeneous contrast enhancement.

On examination, there were bilateral level II neck masses posterior to the angle of the mandible, deep in the sternomastoid muscle. The right neck mass was larger than the left, measuring 5 × 4 cm and 4 × 3 cm, respectively. The masses were well circumscribed and fusiform in shape, firm, not pulsatile and non-tender on palpation. They were movable in a horizontal direction but not vertical. Multiple café-au-lait spots were noted, the largest measuring 15 × 8 cm over the left shoulder. Flexible endoscopy of the larynx revealed mobile bilateral vocal folds. Magnetic resonance imaging (MRI) with gadolinium contrast showed bilateral well-circumscribed, T1 isointense, T2 hyperintense masses in the post-styloid parapharyngeal space (Fig. 1). The right neck mass demonstrated heterogenous contrast enhancement and measured 3.0 × 4.6 × 7.2 cm. It displaced the internal carotid artery medially and partially compressed the internal jugular vein on the right side. No splaying of carotid arteries was seen on a magnetic resonance angiogram.

Considering the clinical and imaging features, we concluded that the masses were parapharyngeal neurogenic tumors, most likely neurofibromas arising from the vagus nerve or cervical plexus. The patient expressed a desire to have the right neck mass removed, given concerns about the progressive increase in size causing worsening compressive symptoms and the cosmetic appearance of the mass. After a thorough discussion with the patient, during which he was informed about possible postoperative functional deficits, a surgical exploration, with excision of the right neck tumor was planned, with the intention to preserve nerve function. The patient consented to nonselective laryngeal reinnervation and transoral injection laryngoplasty.

**Figure 2:**
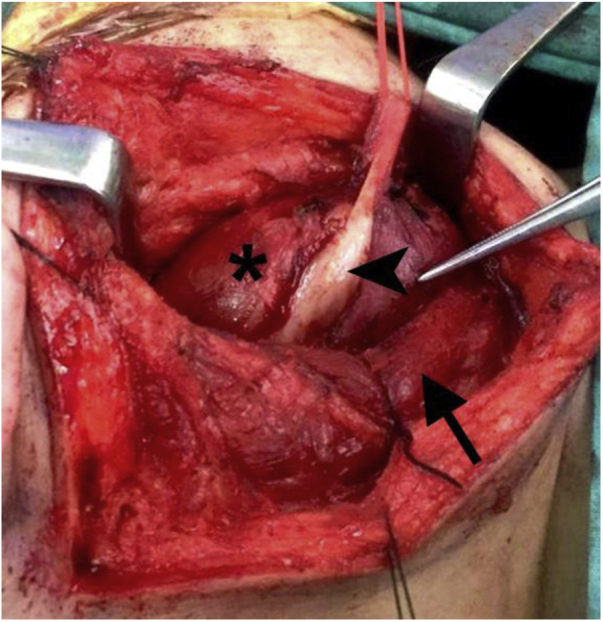
The tumor (*) located deep to sternomastoid muscle (arrow), attached to hypoglossal nerve (arrowhead).

**Figure 3:**
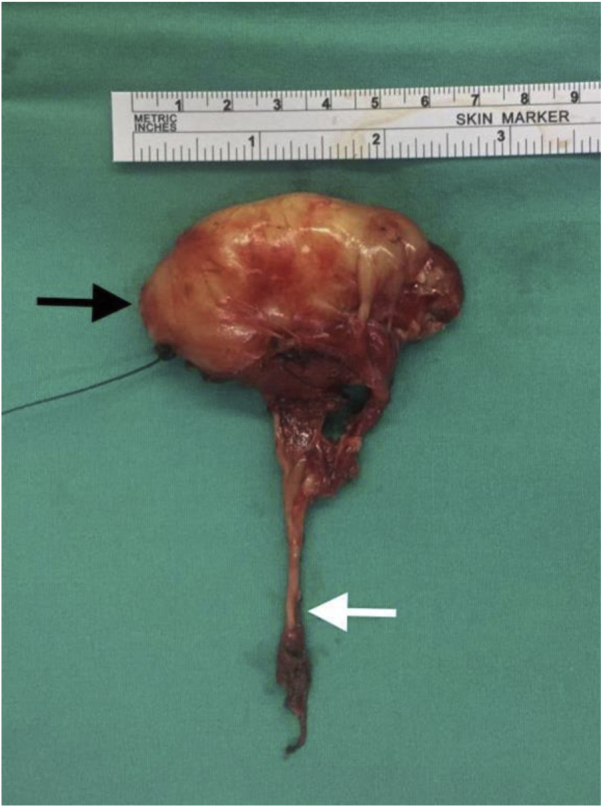
The tumor had engulfed the whole proximal end of the hypoglossal nerve (white arrow). Black arrow shows the cranial end of the tumor.

Intraoperatively, the mass was located in the parapharyngeal space, deep in the internal jugular vein, displacing the carotid artery medially. It appeared well encapsulated, yellowish in color and measured 7 × 5 × 3 cm. The superior end of the mass was located approximately 2 cm below the base of the skull. The inferior end of the tumor was attached to the hypoglossal nerve where it crossed the internal and external carotid arteries (Fig. 2). The vagus nerve was adherent to the tumor but was separated with gentle dissection. Therefore, the plan for nonselective laryngeal reinnervation and injection laryngoplasty was abandoned. The internal and external carotid arteries and internal jugular vein were freed from the tumor. The hypoglossal nerve distal to the mass appeared swollen and abnormal. The proximal part of the hypoglossal nerve was completely engulfed in the tumor (Fig. 3). Due to the difficult location of the mass near the base of the skull, it was not possible to delineate the hypoglossal nerve fascicles from the tumor, and the nerve had to be sacrificed and excised, together with the tumor.

**Figure 4:**
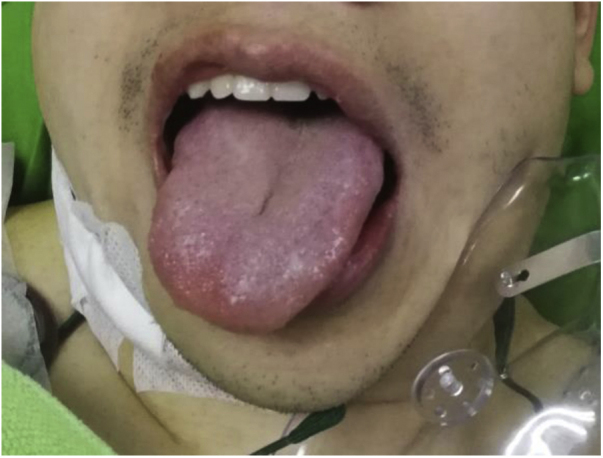
The patient developed right hypoglossal palsy immediate post-operation.

Immediately post-surgery, the patient developed right hypoglossal palsy. His tongue deviated to the right side on protrusion (Fig. 4). There was no dysphagia or slurred speech. On day 3 post-surgery, he developed hoarseness and coughing while drinking. Flexible endoscopy of the larynx revealed an immobile right vocal fold with bowing in the lateral position. There was a large glottic gap on phonation, and laryngeal sensations were reduced as evidenced by pooling of saliva in the larynx and hypopharynx. Augmentation of the right vocal fold by injection laryngoplasty with hyaluronic acid was performed. The hoarseness and aspiration symptoms improved after the procedure.

**Figure 5:**
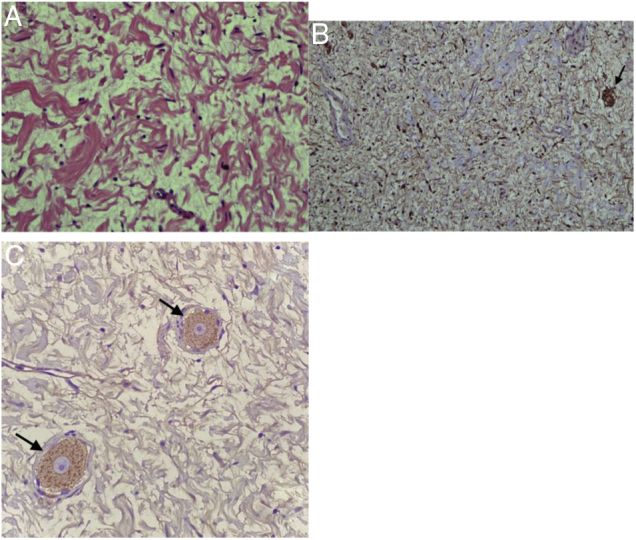
(A) The tumor cells display fine granular chromatin, buckled nuclei with a narrow tapering outline, inconspicuous nucleoli, and poorly defined pale cytoplasm (H&E, ×100). (B) Spindle cells and mature ganglion cells (arrow) are positive for S100. (C) Mature ganglion cells (arrow) are positive for synaptophysin.

The histopathology report of the neck tumor revealed features typical of a neurofibroma, without malignant degeneration (Fig. 5a). The spindle cells expressed S100 protein diffusely, and mature ganglion cells showed reactivity for S100 and synaptophysin (Fig. 5b and c). At the 1 month followup, the right vocal fold remained immobile. However, clinically the patient’s voice quality had improved, and aspiration symptoms were markedly improved.

In view of the left neck mass showing similar clinical and radiological features to the right mass, it is highly possible that it shares a similar neural origin with the contralateral side. After consultation with the patient, he was not in favor of excision of the left neck mass in the near future, considering the potential sequelae of bilateral hypoglossal palsies. He remains under followup, with expectant management and a plan for repeat imaging to reassess the progression of the tumor.

## Discussion

Neurofibromatosis, a genetic disorder characterised by abnormalities of the skin, bone and nervous system, is associated with the development of neurogenic tumors throughout the body. Neurogenic cells originate from neural crest cells, which give rise to schwannomas and neurofibromas.^4^ Patients with neurofibromatosis have variable phenotypic expression, exhibiting a spectrum of clinical manifestations and severity of disease.

Neurogenic tumors in the neck can originate from the lower four cranial nerves, cervical sympathetic nerve, cervical or brachial plexus. A neurogenic tumor of the vagus nerve is extremely uncommon. A retrospective single center study showed that only 5% (2/42) of neurogenic tumors arose from the hypoglossal nerve.^4^ The majority of neoplasms of the hypoglossal nerve are schwannomas rather than neurofibromas.^2^, ^5^ Of the few reported cases of hypoglossal neurofibromas in the literature, all were reported to arise from the cranial part of the nerve or terminal part in the tongue.^5^ A neurofibroma arising from the cervical part of the hypoglossal nerve is extremely rare. There are only two case reports of a cervical hypoglossal nerve neurofibroma in the English literature, both of which were reported more than 80 years ago. To the best of our knowledge, this is the first report of bilateral neurofibromas originating from the cervical part of hypoglossal nerves in a patient with NF-1.

MRI is the optimum imaging modality for the diagnosis of nerve sheath tumors. However, it may be difficult to determine the origin of such tumors based on imaging alone. High-resolution MR neurography can delineate neural fascicles in relation to the tumor, which helps to inform preoperative planning and the appropriate surgical approach.^3^ The authors of a previous study proposed that MRI with diffusion tension imaging, which allows direct visualisation of specific nerve tracts entering and leaving a peripheral nerve sheath tumor, is useful for anatomic and functional evaluation of peripheral nerve lesions.^6^

Fine needle nspiration (FNA) has a limited role in distinguishing among the various types of cervical neurogenic tumors.^3^ The diagnosis of a neurofibroma in the present case was evident based on the patient’s underlying disease and typical imaging features, making FNA non-essential. Although FNA may be useful to differentiate benign and malignant lesions, previous research reported that only 60% of FNA yielded adequate material for interpretation.^7^ A biopsy was not recommended in cases of neurofibromas, as it tended to cause bleeding and scarring inside and around the tumors.^5^

The decision for surgery in patients diagnosed with a neurofibroma should take into account the benefits and risks, including preoperative symptomatic severity and predicted postoperative neurological deficits. Despite the difficulty in determining the nerve of origin in cervical neural tumors, thorough examinations must be performed before surgery to assess and document the neural function. All patients must be informed about the risk of post-treatment functional deficits and sequelae. In the present case, hemiglossal paralysis was a consequence of resection of the tumor that originated from the hypoglossal nerve. Ipsilateral vocal fold immobility on day 3 most likely resulted from temporary dysfunction due to manipulation of the vagus nerve during dissection of the tumor. Intraoperative vagus nerve integrity monitoring may help to identify disruption of nerve function intra-operatively. If a vagus nerve deficit is detected, rehabilitation by nonselective reinnervation or injection laryngoplasty can be done during surgery.^8^

There are several factors to consider in the management of a neurofibroma in patients with NF-1 as compared to sporadic cases. Some authors have proposed watchful waiting in NF-1 cases, as 54% of patients with NF-1 have more than one neurofibroma.^1^ Surgery may be considered when a patient is symptomatic or when there is a suspicion of malignant transformation.^1^ Other authors have suggested early surgical interventions in symptomatic neurofibromas, as 50%–60% of malignant peripheral nerve sheath tumors are associated with NF-1.^3^ In one study, the lifetime risk of malignant transformation in NF-1 patients was reported to be 1%–2%.^4^ Another study reported a significantly higher risk of 8%–13%.^1^ The risk was significantly greater in patients with a history of ionising radiation, long-standing disease and the presence of multiple neurofibromas from an early age. Close monitoring and a high level of suspicion in the presence of a rapidly enlarging and painful swelling are essential for early detection of lesions with malignant transformation.

Excision of a neurofibroma carries a risk of neural dysfunction because of its neural origin. Nevertheless, surgery remains the treatment of choice because neurofibromas are relatively radioresistant.^7^ There have been some attempts to achieve gross tumor resection with preservation of neural function. Although some authors advocated the use of intra-capsular enucleation for nerve sheath tumors, postoperative nerve palsy occured in 16%–40% in the study.^7^ There is also a risk of recurrence in the presence of residual tumor in the capsule after surgery. However, the previous studies mainly focused on extracranial schwannomas. A neurofibroma, in contrast to a schwannoma, contains all the elements in a nerve, namely axons, sheath cells and connective tissue. Neurofibroma usually involves multiple fascicles of the original nerve that are indistinguishable from the surrounding nerve tissue, thus making complete excision a challenge without sacrificing much of the nerve.^6^, ^9^

On the other hand, some authors reported complete excision in 79% of NF-1 patients with neurofibromas, with no deterioration of neural function.^3^ In our case, the hypoglossal nerve was grossly engulfed by the tumor and thus resected, together with the tumor. Nerve reconstruction was not performed, as the proximal stump was high up in the base of the skull. Tandon et al. suggested a transcervical approach with a paramedian mandibulotomy in cases of tumors that extended to the base of the skull or exceeded 8 cm in diameter.^6^ Another study reported that a cranial base approach would provide sufficient exposure for intracranial dumbbell-shaped hypoglossal nerve sheath tumors.^10^

## Conclusion

Hypoglossal nerve neurofibromas are uncommon, particularly those that arise from the cervical part of the nerve. We describe a patient with NF-1 and rare parapharyngeal hypoglossal nerve neurofibroma. A surgical intervention was indicated, as the patient was symptomatic due to progressive enlargement of the tumor and the increased risk of malignant transformation. Gross tumor resection with preservation of neural function is the treatment of choice for nerve sheath tumors. However, this can be difficult to achieve due to the nature of neurofibromas, which involve multiple nerve fascicles, and their locations near the base of the skull. More studies are needed to improve the treatment outcome of cervical hypoglossal nerve neurofibroma, which is a rare clinical entity.

## Conflicts of interest

The authors declare no conflicts of interest.
